# FeAture Explorer (FAE): A tool for developing and comparing radiomics models

**DOI:** 10.1371/journal.pone.0237587

**Published:** 2020-08-17

**Authors:** Yang Song, Jing Zhang, Yu-dong Zhang, Ying Hou, Xu Yan, Yida Wang, Minxiong Zhou, Ye-feng Yao, Guang Yang

**Affiliations:** 1 Shanghai Key Laboratory of Magnetic Resonance, East China Normal University, Shanghai, China; 2 Department of Radiology, The First Affiliated Hospital with Nanjing Medical University, Nanjing, China; 3 MR Scientific Marketing, Siemens Healthcare, Shanghai, China; 4 Shanghai University of Medicine & Health Sciences, Shanghai, China; Cardiff University, UNITED KINGDOM

## Abstract

In radiomics studies, researchers usually need to develop a supervised machine learning model to map image features onto the clinical conclusion. A classical machine learning pipeline consists of several steps, including normalization, feature selection, and classification. It is often tedious to find an optimal pipeline with appropriate combinations. We designed an open-source software package named FeAture Explorer (FAE). It was programmed with Python and used NumPy, pandas, and scikit-learning modules. FAE can be used to extract image features, preprocess the feature matrix, develop different models automatically, and evaluate them with common clinical statistics. FAE features a user-friendly graphical user interface that can be used by radiologists and researchers to build many different pipelines, and to compare their results visually. To prove the effectiveness of FAE, we developed a candidate model to classify the clinical-significant prostate cancer (CS PCa) and non-CS PCa using the PROSTATEx dataset. We used FAE to try out different combinations of feature selectors and classifiers, compare the area under the receiver operating characteristic curve of different models on the validation dataset, and evaluate the model using independent test data. The final model with the analysis of variance as the feature selector and linear discriminate analysis as the classifier was selected and evaluated conveniently by FAE. The area under the receiver operating characteristic curve on the training, validation, and test dataset achieved results of 0.838, 0.814, and 0.824, respectively. FAE allows researchers to build radiomics models and evaluate them using an independent testing dataset. It also provides easy model comparison and result visualization. We believe FAE can be a convenient tool for radiomics studies and other medical studies involving supervised machine learning.

## Introduction

Radiomics extracts a large number of quantitative features from the regions of interest (ROIs) in medical images and maps them to clinical conclusions with machine learning (ML) [1, 2]. Radiomics utilizes not only shape, intensity (first-order), and texture (second-order) features from the original image, but also higher and more abstract features such as those in the wavelet transform domain [3]. While features based on shape, intensity, and texture can be easily compared to observations of radiologists, abstract features often reveal hidden information in the image and may be used to explain the intuition of experts. Radiomics models can be integrated into computer-aided diagnosis (CAD) systems [4] to analyze medical images, and even resolve uncertain cases.

Establishing radiomics models involves supervised learning, which is a subclass of machine learning. Most works of radiomics belong to a binary classification problem [5], which is usually a primer project about radiomics. A typical radiomics study includes the following steps: 1) The dataset is split into two parts: a training dataset used to develop the model, and an independent test dataset used to evaluate the model. 2) The training dataset is further split into a real training dataset and a validation dataset. The real training dataset is used to develop a model and update the parameters (e.g., the slope and intercept of a linear model), and the validation dataset is used to determine the hyperparameters (e.g., a linear model or quadratic model). When the size of the training dataset is limited, cross-validation is often used instead of an independent validation dataset. 3) Each feature is normalized to avoid the effect of different scales [6]. 4) Feature selection and dimension reduction are used to reduce the number of features and keep only those most relevant to the diagnosis to prevent overfitting [7]. 5) A classification or regression model is trained with selected features and the label. 6) The performance of the model on the validation dataset is evaluated using typical clinical statistics such as the area under the receiver operating characteristic (ROC) curve (AUC) [8]. 7) Steps 3 to 6 are repeated with different hyperparameters (e.g., different classifiers). 8) The model with the best performance on the validation dataset is determined, and its performance is evaluated on the independent test dataset.

In the above process, there are many candidate algorithms in each step. To find the optimal model, researchers often need to explore different combinations of algorithms, which can be tedious and may require expertise in machine learning. To address this problem, we designed an open-source software package called FeAture Explorer, which can be used in radiomic studies. Researchers can use FAE to develop models with different combinations of feature normalization methods, feature selection algorithms, and classifiers. FAE can automatically try out all specified combinations of different algorithms and compare the performances of the established models on the validation dataset. Further, in radiomics studies, researchers usually focus on the interpretation of the features in the final built model. FAE also provides common clinical statistics and visualization of models, which helps to facilitate the comparison of different models.

## Materials and methods

### Software design

**Fig 1:**
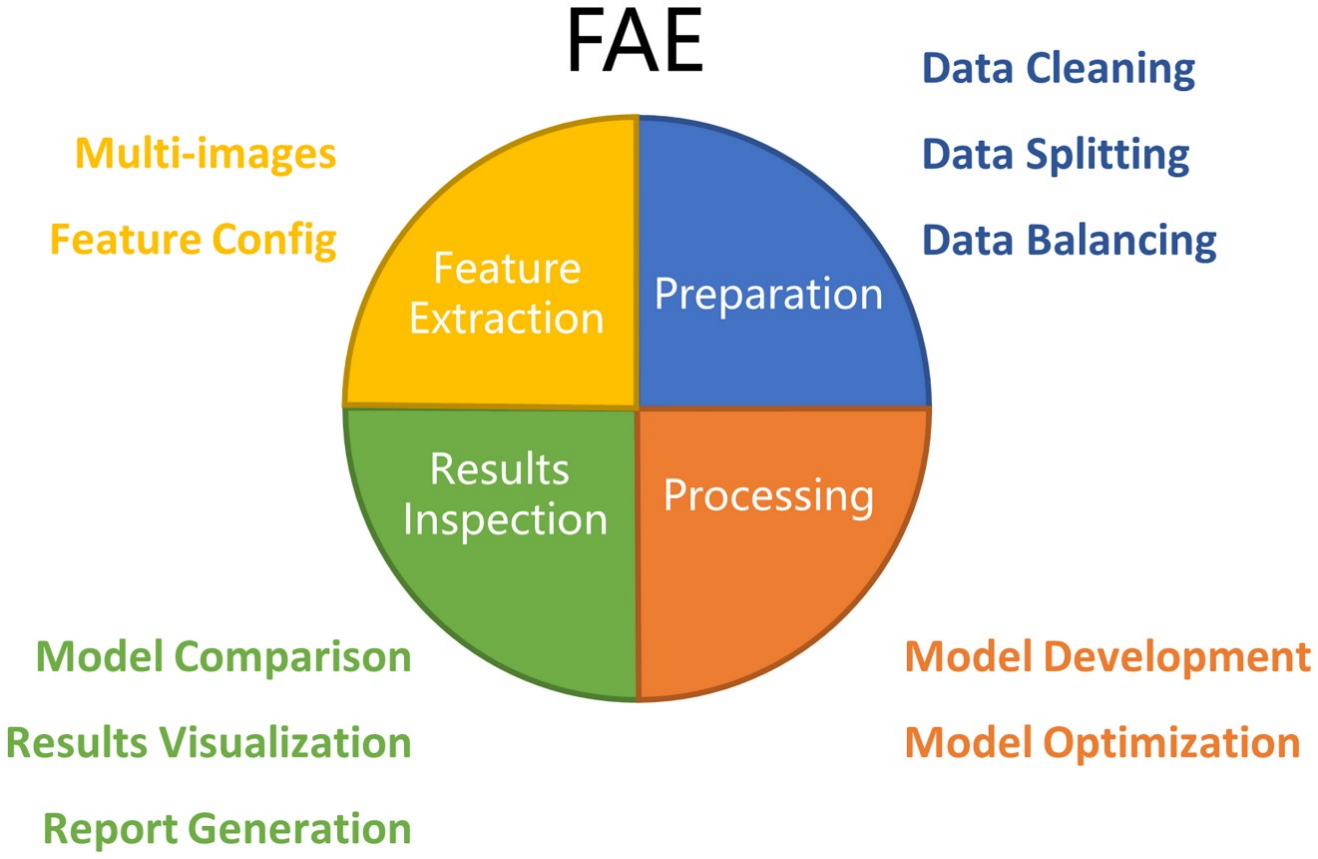
Architecture of FAE. Major functions of FAE include *Feature Extraction*, *Data Preparation*, *Model Development*, *and Result Inspection*.

FAE has four major functions: Feature Extraction, Data Preparation, Model Development, and Results Inspection (Fig 1).

#### Feature extraction

**Fig 2:**
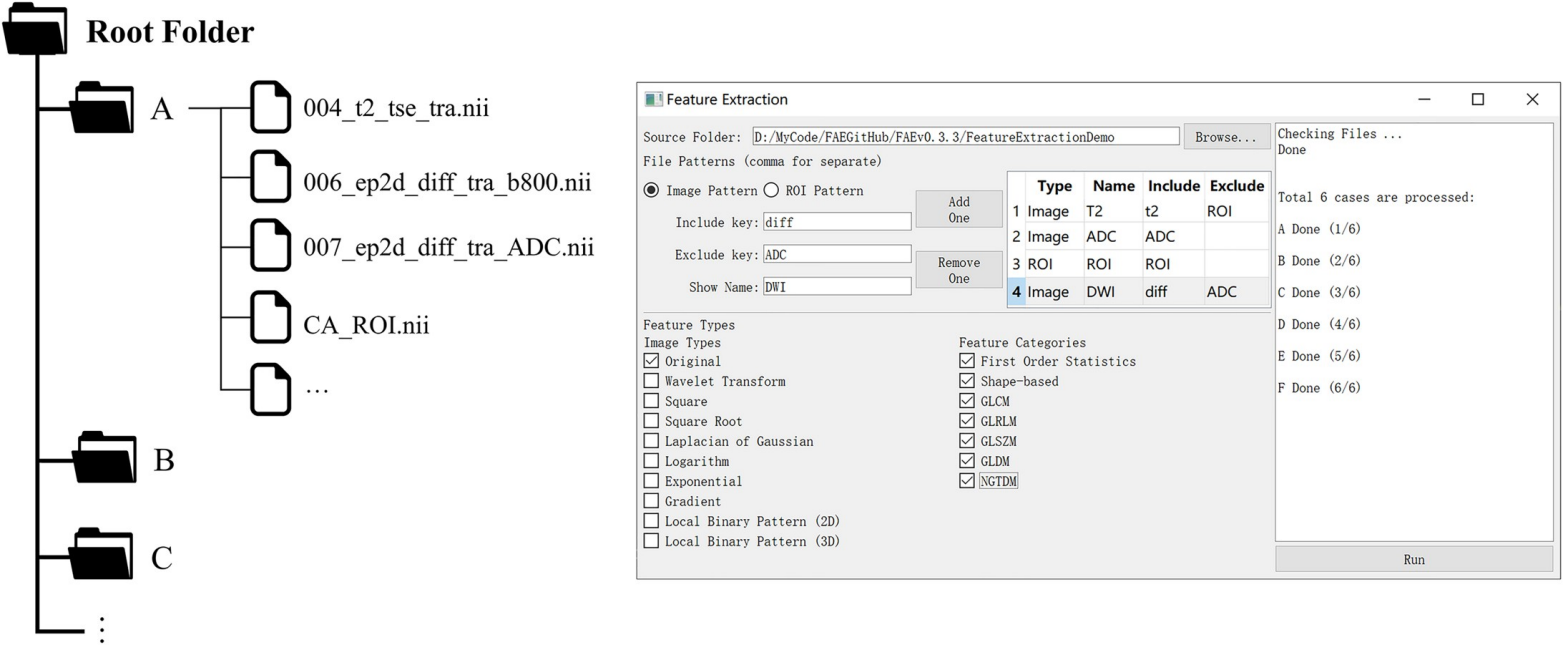
Folder structures and the user interface for feature extraction. For each case, images and the corresponding ROIs were stored in a sub-folder. FAE can be used to extract different kinds of features from different kinds of images conveniently.

Pyradiomics, an open-source module for radiomics feature extraction, was used in FAE [9]. Users need to save the images and the corresponding ROIs in NFITY format and store the files for each case in a separate subfolder in a root folder (Fig 2), before FAE can be used to extract radiomics features for all the cases in a batch. Features from multiple images, including transformed images, such as wavelet, log images, can also be extracted with the same ROIs.

#### Data preparation

**Fig 3:**
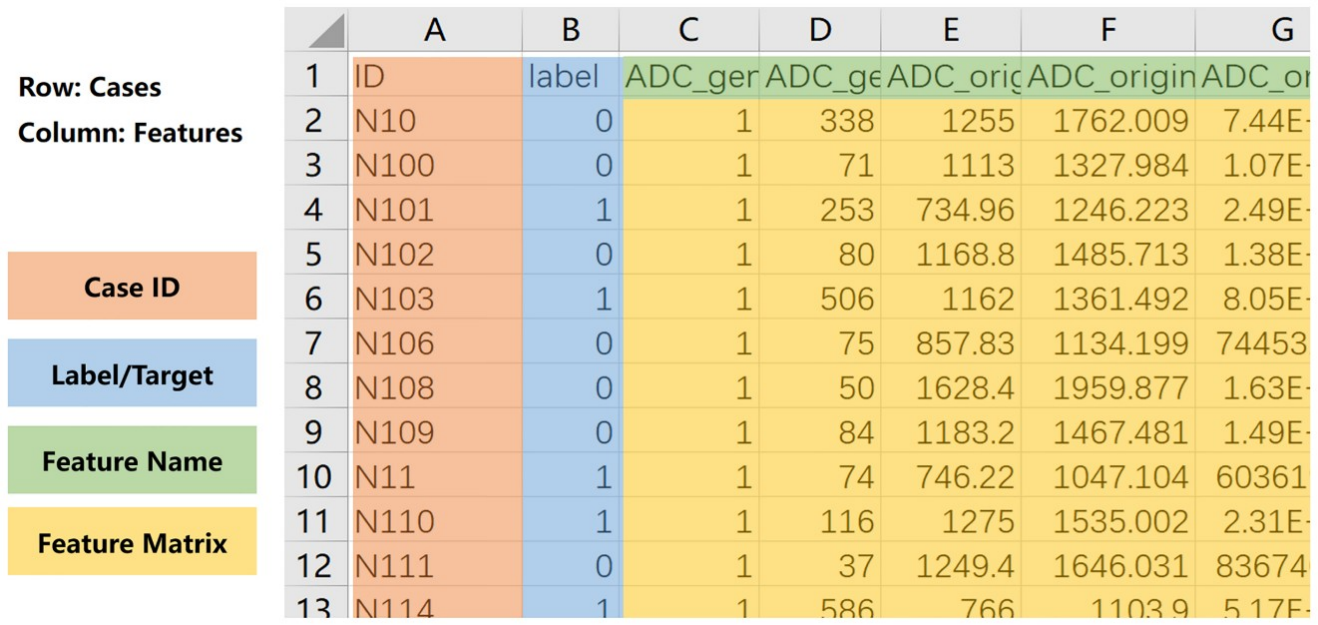
Format of feature matrix used for FAE. Case index is saved in first column and label in second column. Feature names are stored in first row. File should be saved in comma-separated values (CSV) format.

To use FAE, one must prepare a comma-separated values (CSV) file containing a feature matrix in the following format (Fig 3): 1) Each row contains one case, and each column denotes one feature. 2) The first row contains the names of the features, and the first column contains index of the cases. 3) There should be one column with the header of “label” to denote the clinical conclusions. After the feature matrix is loaded, FAE checks the validity of the feature values. FAE can export a log file to help users see which cases and features have invalid values, such as null or text. The features or cases including invalid values can also be removed by one-click in FAE. It should be noted that if a feature is categorical instead of numeric (e.g., gender or tumor stage), the user needs to encode it numerically. FAE can also split the dataset into training and test datasets with a specified ratio, and it will maintain the ratio of positive cases to negative cases constant in the two datasets. If the positive and negative cases are not balanced in number, then FAE provides downsampling, upsampling, synthetic minority oversampling technique (SMOTE) [10] or SMOTETomek [11] to balance the training dataset.

#### Model development

**Fig 4:**
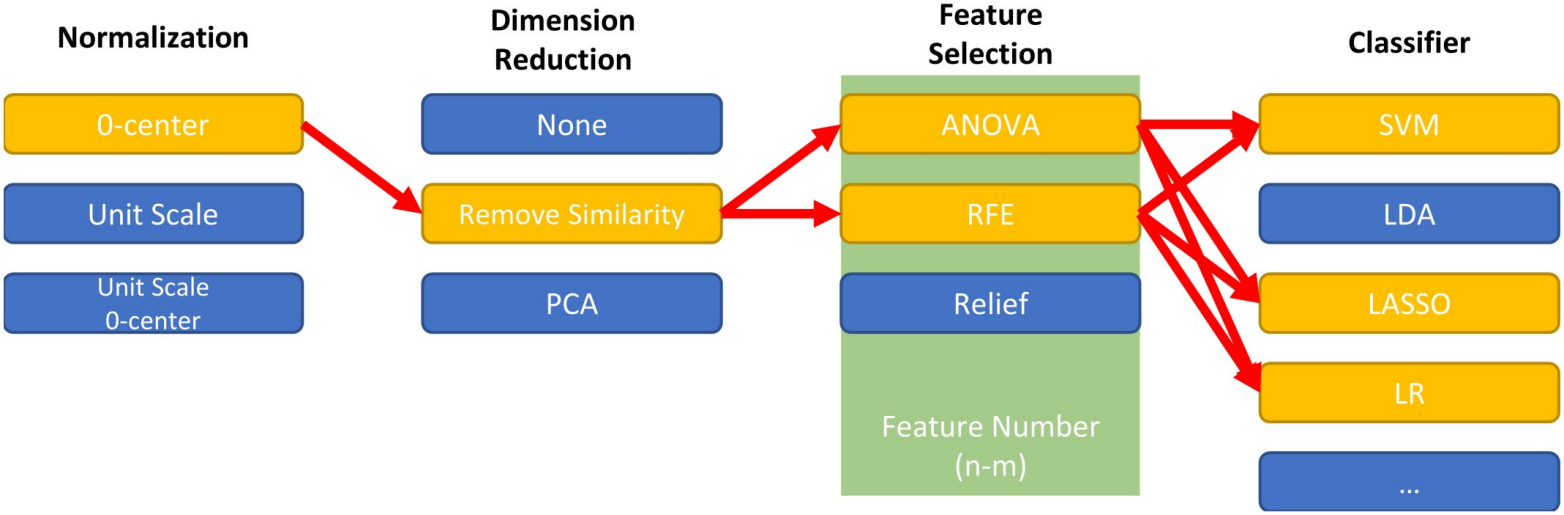
Demo of combination of one radiomics pipeline. For each step of radiomics pipeline, FAE provides some options from which researchers can choose. FAE will try out all possible combinations of selected options using the training dataset.

FAE can help develop a radiomics pipeline that includes the following steps: data normalization, dimension reduction, feature selection, and classifier. Available options for all the components are listed in Table 1. FAE uses all possible combinations of these components to construct radiomics pipelines, train models using the training data, and evaluate the respective results on the validation dataset. For example, if one researcher selects two feature selection methods and three different classifiers, then FAE constructs six pipelines to develop six models (Fig 4). We fix the random seed for each component to make sure the result is reproducible and users can specify any random seed value they prefer for any component in the config file. FAE can be used to grid-search the best hyper-parameters if specified in a config file. For radiomics studies where the dataset size is limited, FAE can use five-fold or ten-fold cross-validation. All models are saved with common clinical statistics and all interim results, which can be loaded later for inspection and allows researchers to use other software to conduct further analysis.

**Table 1:** Available options for each step in the radiomics pipeline: Data normalization, dimension reduction, feature selection, and classifier. All methods implemented based on scikit-learn [12].

Steps	Candidate
Data	Min-max Normalization
Normalization	Z-score Normalization
	Mean Normalization
Dimension	Pearson Correlation Coefficient (PCC)
Reduction	Principle Component Analysis (PCA)
Feature Selection	Analysis of Variance (ANOVA)
	Recursive Feature Elimination (RFE)
	Relief
Classifier	Linear Regression
	Least Absolute Shrinkage and Selection Operator (LASSO)
	Support Vector Machine (SVM)
	Linear Discriminant Analysis (LDA)
	Decision Tree
	Random Forest
	Adaboost
	Gaussian Process
	Naïve Bayes
	Multilayer Perceptron

#### Result inspection

**Fig 5:**
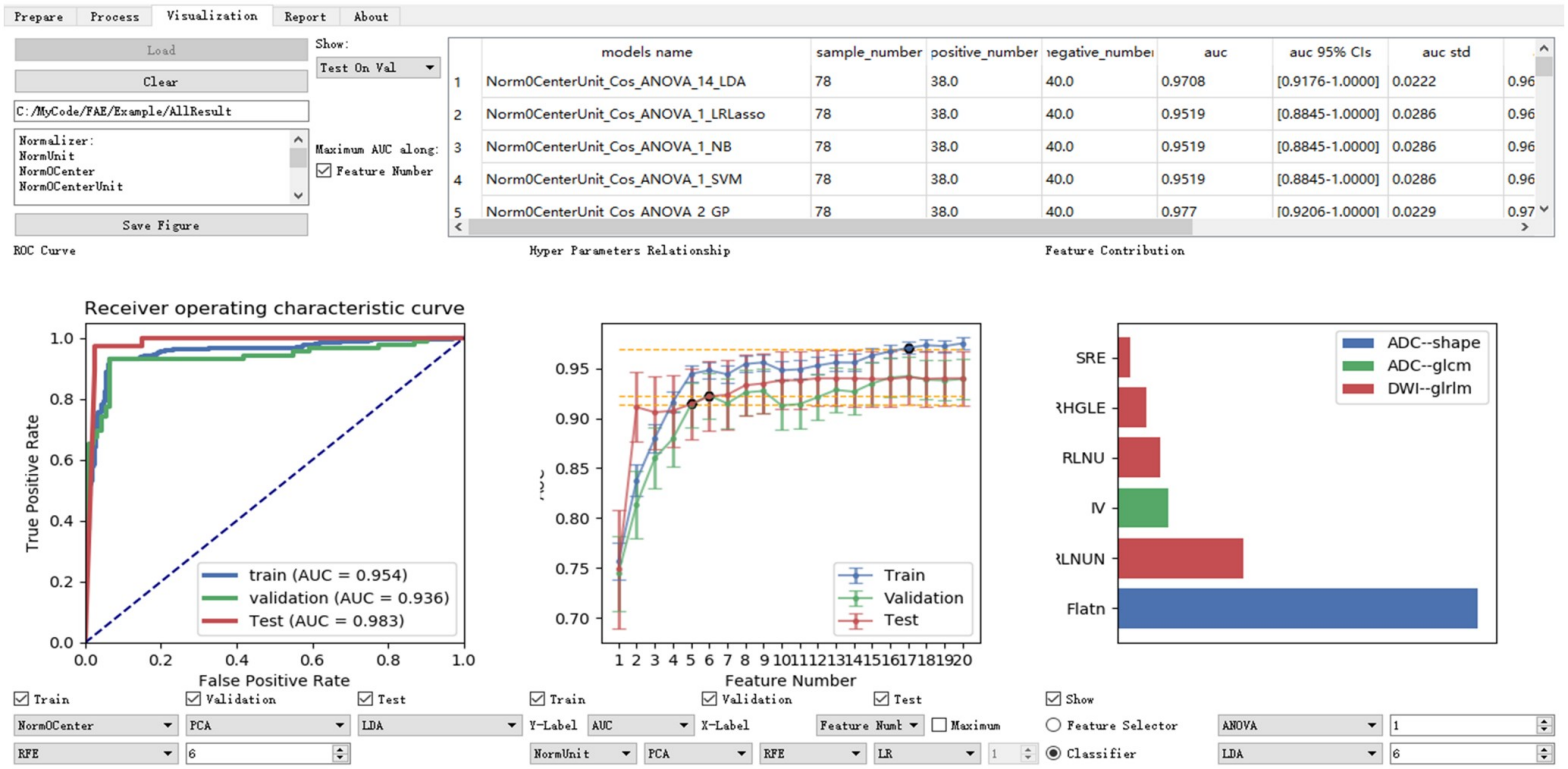
Graphic user interface of *Result Inspection* part of FAE. Common clinical statistics for each model are shown on training dataset and validation dataset. Models are compared using ROC curve, AUC of each model, and feature contributions. FAE can also create high-resolution plots and generate brief description of algorithms used in selected model.

FAE lists common clinical statistics and displays typical figures for model evaluation (Fig 5). The statistics presented include the AUC, 95% confidence intervals of AUC, accuracy, sensitivity, specificity, and others for the training dataset and validation dataset. Statistics for all models are ordered by the AUC on the validation dataset, and thus different models can be compared easily to find the best model. To help the researcher to select right number of features in the final model and to gain a better understanding of the model, FAE shows a plot of AUC against the number of selected features together with the weights of each feature in the model.

FAE was developed in Python 3.6 (https://python.org) and a Qt framework. Most algorithms in FAE were implemented with scikit-learn 0.19 (https://scikit-learn.org) [12]. The source code is openly available on GitHub (https://github.com/salan668/FAE.git).

### Retrospective experiments

To demonstrate the usability of FAE, we used it to differentiate clinically significant prostate cancer (CS PCa) and non-CS PCa (NCS PCa) using radiomics features of lesions in multiparametric magnetic resonance imaging (mp-MRI). We selected 185 cases from the PROSTATEx dataset [13], a public prostate cancer dataset containing 68 CS and 184 NCS PCa. We used the T2W image (TSE, 0.5 × 0.5 × 3.6 mm^3^), diffusion weighted images (DWI, SSEP, 2 × 2 × 3.6 mm^3^) with b-value = 800 s/mm^2^, and the corresponding apparent diffusion coefficient (ADC) maps for feature analysis, which conform to the recommendation of prostate imaging–reporting and data system (PI-RADS). One radiologist with 11 years of clinical experience (Y. Z.) drew the boundary of lesions on T2W transverse images using biopsy results together with ADC and DWI images as reference. We used Elastix (http://elastix.isi.uu.nl/index.php) to align the DWI and ADC images to the T2W images [14].

## Results

We prepared the MR images and used FAE to extract quantitative features from these three series. Types of features extracted included the shape, histogram (intensity-based or first-order), gray level co-occurrence matrix (GLCM), gray level run length matrix (GLRLM), and gray level size zone matrix (GLSZM). From each PCa ROI, 264 features were extracted.

We loaded the feature matrix in the *Data Preparation* page in FAE and split the dataset into a training dataset of 177 samples (CS-PCa/NCS-PCa = 48/129) and an independent test dataset of 75 samples (CS-PCa/NCS-PCa = 48/129). In the *Model Development* page of FAE, we designed the pipelines of radiomics models. We normalized the dataset by *Z-score Normalization*, which subtracts the mean value and divides the standard deviation for each feature. To reduce the dimensions of the row space of the feature matrix, we used a *Pearson Correlation Coefficient (PCC)* for each pair of two features [15]. If the PCC was larger than 0.9, we removed one of them randomly in FAE. We chose the Analysis of Variance (ANOVA), Relief [7], and Recursive Feature Elimination (RFE) for feature selection. The range of the feature number was set from 1 to 20. We also tried a Support Vector Machine (SVM) [16], Linear Discriminative Analysis (LDA) [5], Random Forest [17], and Logistical Regression (LR) [18] to find the best model. The performance of all pipelines was evaluated using five-fold cross-validation.

**Fig 6:**
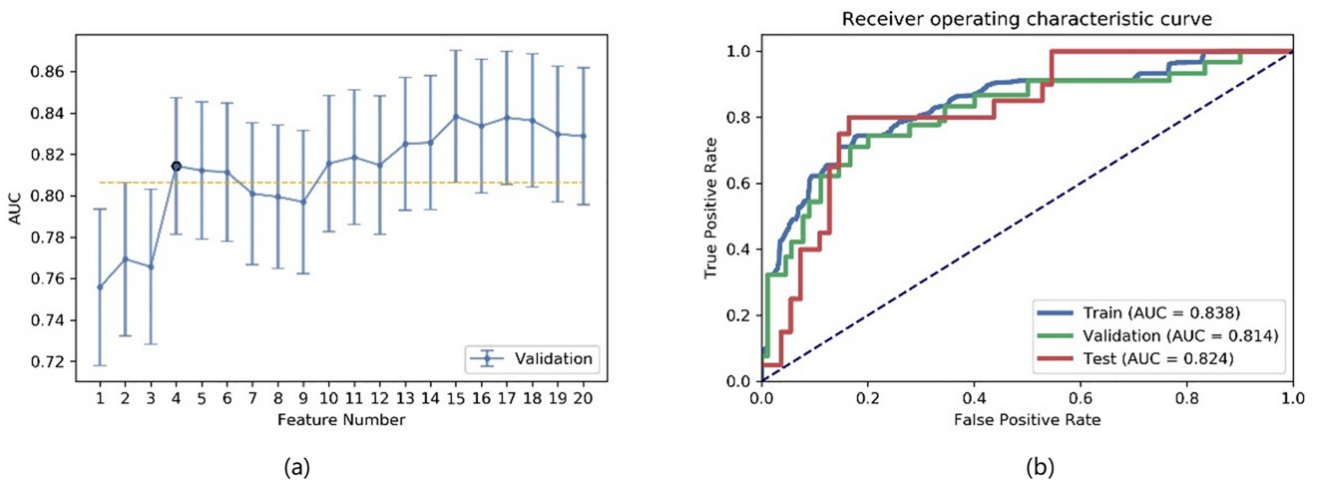
Performance of model generated by FAE. (a) FAE suggested a candidate four-feature model according to “one-standard error” rule, and (b) ROC curves of this model on different datasets.

We compared the AUC of all pipelines on the validation dataset with FAE. The pipeline using ANOVA feature selection and an LDA classifier yielded the highest AUC using 15 features. When “one-standard error” rule was used, FAE also produced a simpler model with only four features [Fig 6(a)] [5] whose ROC curves are shown in Fig 6(b). The AUCs of the training/validation/test datasets achieved 0.838/0.814/0.824, respectively.

Features selected by FAE were the gray level variance of the gray level size zone matrix (GLSZM) from DWI (*GLV*_*DWI*_), interquartile range of the histogram from DWI (*IR*_*DWI*_), autocorrelation of the gray level cooccurrence matrix (GLCM) from DWI (*AC*_*DWI*_), and 10% percentage point of the histogram from ADC (10*Per*_*ADC*_).

**Fig 7:**
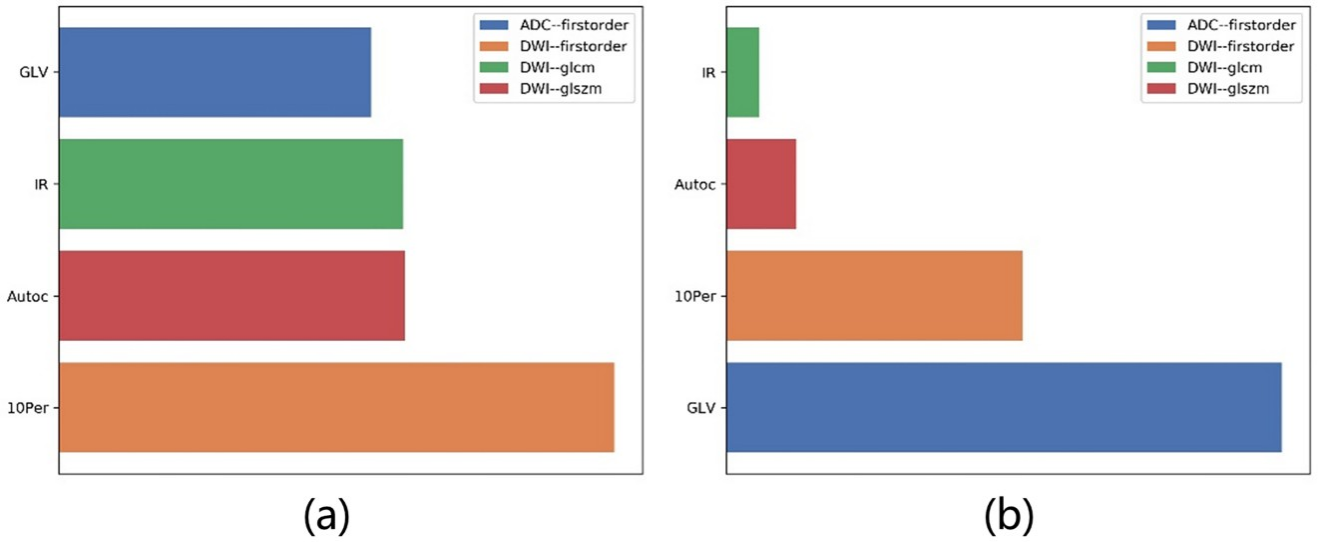
Contribution of features in final model. F-values of selected features by (a) ANOVA, and (b) coefficients of these features in LDA model. Order of contributions of these features is different in ANOVA and LDA.

In the feature selection step, we sorted the features according to their F-values by ANOVA. The F-values of four selected features were *GLV*_*DWI*_ = 72.91, *IR*_*DWI*_ = 45.47, *AC*_*DWI*_ = 45.22, and 10*Per*_*ADC*_ = 41.03. We used these four features to develop an LDA model. The coefficients were 10*Per*_*ADC*_ = 7.98, *IR*_*DWI*_ = −1.89, *AC*_*DWI*_ = 0.95, and *GLV*_*DWI*_ = 14.96. The F-values and their contributions are shown in Fig 7(a) and 7(b). We found that the order of the contributions of the features was different in ANOVA and LDA.

**Fig 8:**
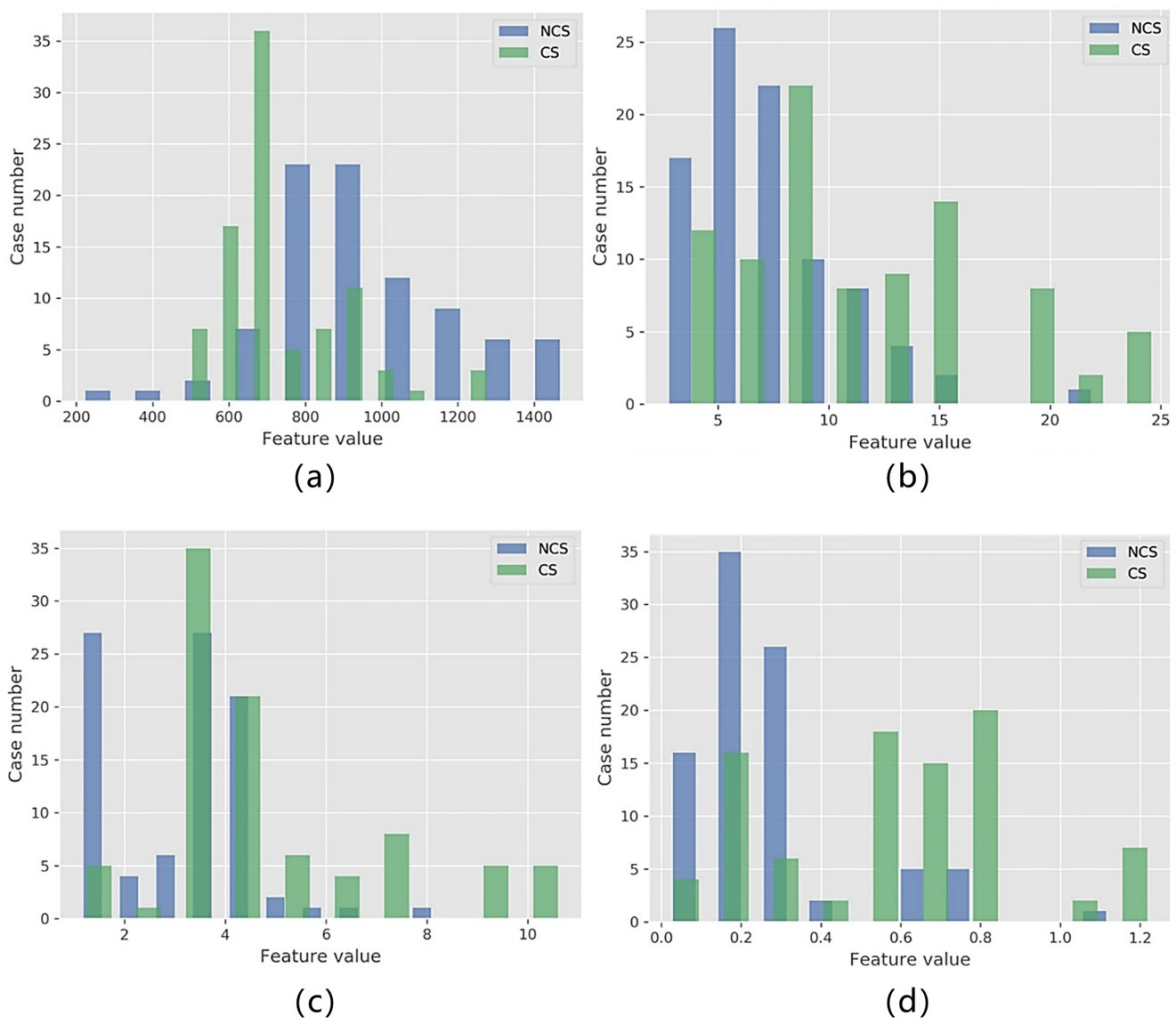
Histograms of selected features in CS PCa and NCS PCa. Distribution of *GLV*_*DWI*_, *IR*_*DWI*_, *AC*_*DWI*_, and 10*Per*_*ADC*_ shown as (a) through (d), respectively.

The histograms of each selected feature in the two classes are shown in Fig 8. While no single feature can be used to classify the CS and NCS PCa perfectly, each feature has different distributions in the two classes. This explains why we can combine these four features to establish a good model.

## Discussions

We developed an open-source software package named FeAture Explorer to facilitate model development and feature exploration in radiomics studies. FAE can be used to process the feature matrix, develop different models, and visualize the results conveniently. For each model, FAE provides a number of statistics together with typical diagrams in which radiologists might be interested. FAE can also produce a brief description of the model, including its predictive ability, components in the pipeline, and features selected.

We demonstrated the use of FAE using CS/NCS PCa differentiation on the PROSTATEx dataset. We tried 12 different pipelines (combining 3 feature selection methods and 4 classifiers) and compared their AUCs on the validation dataset to find the optimal model. Then, FAE was used to find a more robust model that used four features but achieved a comparable AUC. We used an independent testing dataset to evaluate the performance of the final model. The AUC of 0.824 on the test dataset is comparable to that in the work of L. Wang [19]. The AUC of the model on the test dataset was close to those on the training and validation datasets, implying that the model was neither overfitting nor underfitting.

FAE can be used not only for radiomics studies but also for supervised binary classification problems. Since all processors (algorithms) in the FAE pipeline are independent of each other, researchers can add their own algorithms to construct specific pipelines. In addition, since FAE saves all intermediate results in the pipelines, researchers can process these intermediate results with other software. For instance, researchers often compare the model built with only clinical features and the model with image features. FAE can build the radiomics model and export selected radiomics features. Then we can combine them with the clinical features for model development [20]. Moreover, models developed with FAE can be combined with the ensemble method [21].

We used pyradiomics in FAE to extract image features. However, we did not integrate a UI for ROI labeling. ROI labeling requires real time interaction and is often compute-intensive when semi-automatic segmentation algorithms are involved, where C and C++ programs are more suitable. Some free (e.g., 3D Slicer) or commercial (e.g., Frontier of Siemens Healthcare) software packages can be used for ROI labeling. While some of them can also be used for feature extraction, FAE is more efficient because it can extract all desired features from all cases and combine them into a feature matrix in one click.

There are several limitations of FAE as follows: 1) In machine learning, the hyperparameters of the feature selection and classifiers have an important effect on the performance of the model [5]. While FAE provides an option to tune hyper-parameters with a config file, it lacks a user-friendly UI for hyper-parameter tuning. 2) FAE can be used conveniently for binary classification, but it has not provided an integrated UI for multi-label classification and regression problems for now. These problems should be addressed in future versions.

## Conclusion

FAE provides a convenient way to develop radiomics models. Radiologists can use FAE to clean the feature matrix, develop different models, compare the performance, and visualize the results for their own radiomics studies. We opened the source code of FAE, and researchers can use it freely and extend it to test new ideas and algorithms at will.
